# Distribution et itinéraire thérapeutique des patients reçus pour accident vasculaire cérébral à l'Hôpital Régional de Bafoussam, Cameroun

**DOI:** 10.11604/pamj.2019.34.174.19928

**Published:** 2019-12-04

**Authors:** Brice-Donald Agokeng Kemnang, Landry Beyala Bita’a, Styve Hermane Simo Yomi, Brice Tajo Djidjou, Romarique Medjou Mboumo, Cyrielle Djouda Douanla, Victorien Wouafack-Kenfack, Franck-Carrel Nguetsa Tsakeng, Olivia Tania Homla Megaptche, Jérôme Ateudjieu

**Affiliations:** 1 Département de Santé Publique, Faculté de Médecine et des Sciences Pharmaceutiques, Université de Dschang, Dschang, Cameroun; 2 Meilleur Accès aux Soins de Santé (M.A. SANTE), Mogode, Cameroun; 3 Meilleur Accès aux Soins de Santé (M.A. SANTE), Yaoundé, Cameroun; 4 Department of Medicine, Faculty of Health Science, University of Buea, Buea, Cameroon; 5 Division de la Recherche Opérationnelle en Santé, Ministère de la Santé Publique, Yaoundé, Cameroun

**Keywords:** Distribution, itinéraire thérapeutique, accidents vasculaires cérébraux, Cameroun, Distribution, therapeutic path, stroke, Cameroon

## Abstract

**Introduction:**

L'incidence des accidents vasculaires cérébraux (AVC) est sans cesse croissante en Afrique. La morbidité et la mortalité liées à cette maladie dépendent de la prise en charge des patients aussi bien en communauté que dans les formations sanitaires (FOSA). L'objectif était de décrire la distribution de l'évolution et l'itinéraire thérapeutique des patients reçus pour AVC à l'hôpital régional de Bafoussam (HRB).

**Méthodes:**

Il s'agissait d'une étude de cohorte non contrôlée ciblant les patients qui ont été diagnostiqués pour AVC. Les données étaient collectées à l'aide de deux outils dont un questionnaire anonyme adressé au patient ou à son garde sur la prise en charge pré-hospitalière du patient et une grille pour la collecte des données sur les documents de suivi du patient pendant son hospitalisation. Les données ont été analysées en décrivant la distribution des fréquences des sources et types de soins sollicités avant et pendant l'hospitalisation ainsi que celle de l'évolution à la fin de l'hospitalisation y compris l'incidence des complications.

**Résultats:**

Au total 46 patients ont été inclus dans cette étude avec un âge moyen de 62 ans. Vingt-sept soit 58,7% étaient des femmes et 37 soit 80,4% étaient référés à d'autres FOSA. Quatre (8,7%) patients avaient pris des produits avant leur hospitalisation dont 2 (4,3%) un produit traditionnel. Trente-six (78,3%) patients avaient des antécédents cardiovasculaires dont 22 cas connus d'hypertension artérielle. A la sortie après 10 jours d'hospitalisation, 32 (69,6%) avaient repris leur autonomie et 5 (10,9%) patients étaient décédés.

**Conclusion:**

Peu de patients recourent aux soins non médicalisés avant l'arrivée à l'hôpital. La plupart bénéficient des soins dans les délais requis mais les taux de complications et de décès hospitaliers restent élevés. Une étude devrait être faite pour déterminer les facteurs contribuant à un taux élevé de complications et de décès chez les patients hospitalisés pour AVC à l'HRB.

## Abstract (en)

**Introduction:**

The prevalence of strokes is continuously growing in Africa. Morbidity and mortality related to this disease depend on patients management in the community and health care facilities (FOSAs). The purpose of this study was to describe the outcome and the therapeutic path of patients with stroke admitted to the Bafoussam Regional Hospital (BRH).

**Methods:**

We conducted a cohort non-controlled study of patients who were diagnosed with stroke. Data were collected using two tools such as an anonymous questionnaire among patients or on their pre-hospital management and a grid for the collection of data on patient's outcome during hospitalization. Data were analyzed by describing the frequency distribution of the sources and types of supports solicited prior to and during hospitalization as well as that of patients' outcome at the end of hospitalization including the incidence of complications.

**Results:**

A total of 46 patients were included in this study, with an average age of 62 years: 27(58.7%) were women and 37 (80.4%) were referred to other FOSAs. Four (8.7%) patients had taken products before their hospitalization; 2(4.3%) patients had taken a traditional product. Thirty-six (78.3%) patients had a cardiovascular history, of whom 22 with high blood pressure. Ten days after hospitalization, 32 (69.6%) patients resumed their autonomy and 5(10.9%) died.

**Conclusion:**

Few patients used non-medical treatments before their arrival to hospital. Most of them underwent care in time, but the rate of hospital complications and deaths were high. A study should be conducted to determine the factors contributing to high rate of complications and death in patients hospitalized for stroke at the BRH.

## Introduction

Un accident vasculaire cérébral (AVC) résulte de l'interruption de la circulation sanguine dans le cerveau, en général quand un vaisseau éclate (AVC Hémorragique) ou est bloqué par un caillot (AVC Ischémique) [1,2]. Le symptôme le plus courant de l'AVC est une faiblesse subite ou une perte de la sensibilité de la face ou d'un membre, la plupart du temps d'un seul côté du corps. Les autres symptômes sont la confusion mentale, la difficulté à parler ou des troubles de compréhension, la baisse de la vision unilatérale ou double, la difficulté à marcher, des vertiges, la perte de l'équilibre ou la coordination, des céphalées sévères inhabituelles, l'évanouissement ou l'inconscience [1]. En général, 70% des AVC, 87% des décès et des années de vie corrigées de l'incapacité liée à un AVC surviennent dans les pays à revenu faible ou moyen [2]. Les AVC représentent la deuxième cause de mortalité dans le monde et en Afrique subsaharienne et sont associés à 10% de la mortalité mondiale [3]. Au Cameroun, la situation est plus préoccupante avec 25% du taux de mortalité au cours d'un mois suivant la survenue de l'AVC [4]. La morbidité et la mortalité liées à l'AVC dépendent de l'accès aux soins curatifs et préventifs chez les personnes présentant les facteurs de risque à la maladie qui sont l'exposition au tabac, le diabète, l'hypertension artérielle, l'obésité et les troubles lipidiques [5]. Au Cameroun, la disponibilité des personnes qualifiées pour la prise en charge des facteurs de risque est limitée et le système de détection des cas n'est pas suffisamment sensible conduisant à une insuffisance ou un retard d'accès des patients à la prévention secondaire des cas [3, 6]. Des stratégies de décentralisation des soins au personnel infirmier ont été proposées mais l'évaluation de l'impact de celles-ci sur la mortalité et la morbidité reste encore attendue [7]. Elle peut avoir un impact limité si dans les formations sanitaires, la disponibilité du matériel de dépistage des cas reste aussi limitée. Les insuffisances soulignées par les études antérieures prédisent fréquence élevée des cas d'AVC au sein de la population camerounaise. Réduire la mortalité et la morbidité nécessite d'identifier et anticiper les faiblesses sur le chemin des cas d'AVC qui ne sont pas rares. La présente étude espère documenter les faiblesses sur les conditions de transport, les délais de prise de décision pour la recherche des soins, les sources de soins et les réponses offertes par ces sources, les attitudes du personnel de santé et des proches des personnes malades quand surviennent les cas d'AVC. Les résultats devraient pouvoir être des sources d'évidence pour proposer les actions de formation, de communication et les interventions pour réduire la mortalité et la morbidité liées à l'AVC et une prise en charge inadéquate des cas.

## Méthodes

**Schéma d'étude**: il s'agissait d'une étude de cohorte non contrôlée à deux volets; le volet rétrospectif collectait par un questionnaire administré au garde malade ou au patient lui-même les données sur la prise en charge pré hospitalière du patient; le volet prospectif collectait à l'aide d'une grille de revue documentaire les données sur le suivi du patient pendant son hospitalisation.

**Figure 1:**
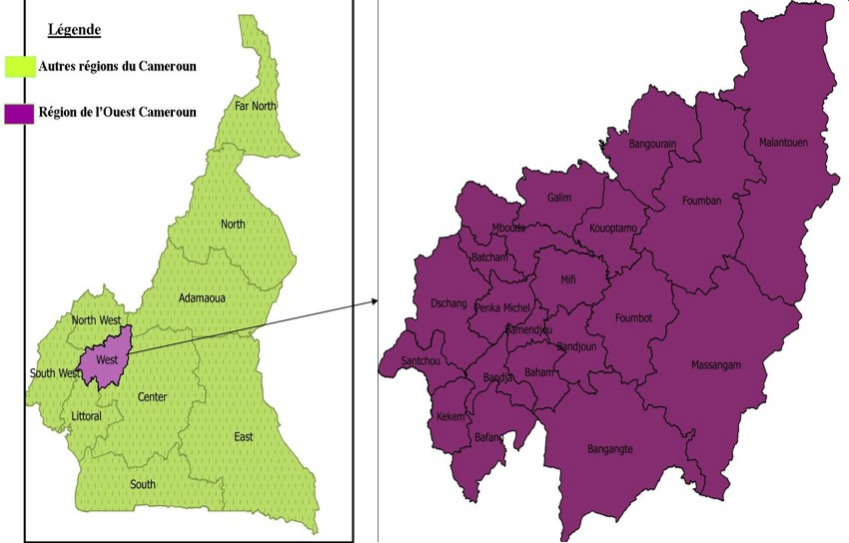
Localisation de la région de l'Ouest Cameroun avec ses Districts de santé

**Lieu de l'étude**: les données ont été collectées auprès des patients reçus dans les services de neuropsychiatrie et de médecine interne de l'Hôpital Régional de Bafoussam, hôpital de référence de la région de l'Ouest Cameroun (Figure 1), situé dans le district de santé de la Mifi; Région à 20 districts de santé avec une population totale estimée en 2018 à environ 2.065.165 habitants.

**Période d'étude**: l'étude s'est déroulée sur la période allant d'octobre 2017 à juillet 2018 soit 10 mois.

### Population d'étude

**Population cible**: tout patient présentant un accident vasculaire cérébral.

**Population source**: patients consultant à l'Hôpital Régional de Bafoussam pendant la période du 02 avril au 30 mai 2018.

**Critères d'éligibilité**: tout patient chez qui le médecin soupçonnait un AVC.

**Critères de sélection**: 1) était inclus dans l'étude tout patient consentant reçu en consultation dans l'un des services de l'HRB ciblés par l'étude et présentant un AVC confirmé au scanner; 2) exclu tout patient consentant qui, à un moment donné refusait de continuer à répondre au questionnaire, ainsi que tout patient qui présentait une menace vitale.

**Méthode d'échantillonnage**: il s'agissait d'un échantillonnage exhaustif des patients victimes d'AVC; les données ont été collectées à partir d'un questionnaire administré aux patients victimes d'AVC ou à leur représentant légal pour la gestion pré hospitalière, d'une grille de revue documentaire pour le suivi hospitalier du patient.

**Gestion des données**: la gestion des données a commencé par le codage des questionnaires par l'équipe en charge de celle-ci. La saisie et l'analyse des données ont été faites à l'aide du logiciel Epi Info version 7.1.3.3. Ses données ont été exportées dans le logiciel Microsoft Excel et à l'aide du complément XLSAT 2018, les fréquences et proportions ont été calculées. Les données ont été analysées en décrivant la distribution des fréquences des sources et types de soins sollicités avant et pendant l'hospitalisation ainsi que celle de l'évolution à la fin de l'hospitalisation, y compris l'incidence des complications et de la mortalité.

## Résultats

Au total 46 patients victimes d'AVC ont été inclus au cours de la période d'étude. Il y avait 27 femmes (58,7%); les patients interviewés étaient âgés de 42 à 88 ans avec une moyenne d'âge de 62 ans (IC à 95%). L'AVC est survenu chez 39 patients de plus de 50 ans (84,9%). Parmi ses patients, 33 (71,3%) vivaient hors de la ville de Bafoussam. La profession la plus représentée était les ménagères avec 23 patients (50%); 27 (58,7%) patients étaient mariés (Tableau 1). Il en ressort que 16 (34,8%) patients avaient déjà perdu connaissance dans le passé sans avoir eu un choc à la tête, dont 7/16 (43,8%) au cours des 6 derniers mois précédant la survenue de l'AVC; 19 (41,3%) avaient déjà eu une faiblesse musculaire d'un des membres ou d'un côté de la face dans le passé; 36 (78,3%) patients avaient des antécédents cardiovasculaires dont 22 patients (47,8%) étaient hypertendus connus et 13(37,1%) diabétiques (Tableau 2).

**Tableau 1:** Description de la population d’étude

Variable	Effectif	Fréquence (%)
**Age**		
Moins de 50 ans	7	15,1
50-60 ans	13	28,3
61-70 ans	13	28,3
Plus de 70 ans	13	28,3
**Sexe**		
Féminin	27	58,7
Masculin	19	41,3
**Lieu de résidence**		
Bafoussam	13	28,3
Hors de Bafoussam	33	71,7
**Niveau d'éducation**		
Non scolarisé	13	28,3
Primaire	10	21,7
Secondaire	17	37
Supérieur	6	13
**Religion**		
Chrétien	30	65,2
Animiste	5	10,9
Pas d'obédience religieuse	11	23,9
**Profession**		
fonctionnaire	10	21,7
Ménagère	23	50
Commerçant(e)	13	28,3
**Statut matrimonial**		
Marié(e)	27	58,7
Célibataire	1	2,2
Veuve/veuf	18	39,1

**Tableau 2:** Antécédents, description et principaux symptômes ressentis par les patients

Variable	Effectif	Fréquence (%)
Patients ayant déjà perdu connaissance dans le passé sans avoir eu un choc à la tête (oui)	16	34,8
**Patients ayant déjà perdu connaissance dans le passé**		
< 1 mois	3	18,8
1 - 6 mois	7	43,8
Patients ayant déjà eu une faiblesse de la force d’un de ces membres ou d’un côté de la face dans le passé (Oui)	19	41,3
Patient souffrant de l’HTA (oui)	22	47,8
Diabète (oui)	13	37,1
**Provenance patient**		
Venu de lui même	9	19,6
Référé	37	80,4
**Circonstances de survenu de l’AVC**		
Dormait	12	26,1
Conduisait	1	2,2
Assis	10	21,7
Exerçait une activité physique	23	50
Patient ayant eu un stress quelconque avant (oui)	25	54,3
**Mode d’installation de l’AVC**	18	39,1
Brutal	18	39,9
Lent	21	45,7
On ne sait pas	7	15,2
Patients ayant convulsé à la survenue de l’AVC (Oui)	3	6,5
Patients ayant perdu connaissance (oui)	40	87
Faiblesse Hémicorps gauche (oui)	27	58,7
Faiblesse Hémicorps droit (oui)	19	41,3
Déviation de la bouche d’un côté (oui)	26	56,5
Asthénie (oui)	42	91,3
Paresthésie (oui)	40	87
Aphasie (oui)	24	52,2
Vertige (oui)	8	17,4
Hyperthermie (oui)	9	19,6
Céphalées (oui)	31	67,4

Des 46 cas d'AVC enregistrés à l'Hôpital Régional de Bafoussam, 9 (19,6%) venaient pour leur première consultation médicale et 37 (80,4%) étaient référés à d'autres FOSA. Il en ressort également que 23 patients (50%) exerçaient une activité physique lors de la survenue des premiers symptômes avec prédominance lente du mode d'installation, soit chez 21 patients (45,7%). Vingt-cinq (25) patients (54,3%) avaient eu un stress quelconque avant de la survenue de l'AVC et 40 (87%) ont perdu connaissance à l'apparition des symptômes (Tableau 2). Les signes et symptômes les plus fréquents ressenti par les patients étaient: la faiblesse Hémicorps gauche chez 27 patients (58,7%); une déviation de la bouche d'un côté chez 26 patients (56,5%); l'asthénie chez 42 patients (91,3%); une paresthésie chez 40 (87%) et des céphalées chez 31 patients (67,4%) (Tableau 2). Le délai entre la survenue des premiers symptômes et la première consultation médicale était entre 1-5h chez 29 patients reçus (63%). Le moyen le plus utilisé pour le transport des patients à l'hôpital était le taxi pour 33 patients (71,7%). L'étude a également montré que 33 patients (71,7%) se trouvaient à une distance supérieure à 5Km de l'hôpital et la durée de transport était entre 1 et 4 heures pour 27 patients (58,7% de); 4 patients (8,7%) avaient reçus des soins avant leur arrivée à l'hôpital dont 2 par une infirmière et 2 par une personne autre qu'un personnel médical. Ces soins étaient des produits pris dont des médicaments chez 2 patients et un produit traditionnel chez 2 autres patients. Il y a eu des freins à leur arrivée à l'hôpital à temps chez 27 patients et le plus observé était l'absence des moyens de transport noté chez 13 patients (48,1%) (Tableau 3).

**Tableau 3:** Gestion du patient après l’apparition des premiers symptômes

Variable	Effectif	Fréquence (%)
**Délai entre la Survenue de l’AVC et la consultation**		
< 1 h	14	30,5
Entre 1 h - 5 h	29	63
>5h	3	6,5
**Moyen de transport emprunté pour l’hôpital**		
Ambulance	1	2,2
Voiture personnelle	8	17,4
Taxi	33	71,7
Moto	4	8,7
Transport à l’hôpital assisté par un personnel médical (Oui)	1	2,2
**Distance entre le lieu de survenu et l’hôpital**		
Entre 1 et 5km	13	28,3
Plus de 5km	33	71,7
**Durée de transport entre le lieu de survenu et l’hôpital**		
< 1 h	19	41,3
1-4 h	27	58,7
Patients ayant reçus des soins avant l’hospitalisation (oui)	4	8,7
Personnel ayant administré ces soins	2	4,3
Infirmier	2	4,3
Personnel non médical	2	4,3
Patients ayant pris un produit avant leur hospitalisation (Oui)	12	26,1
**Nature du produit pris**		
Un médicament	2	4,3
Un produit traditionnel	2	4,3
Frein à l’arrivée à l’hôpital à temps (Oui)	27	58,7
**Différents éléments constituant de frein**		
Problème financier	1	2,2
Absence de moyen de transport	13	28,3
Patient isolé lors de la survenue de l'AVC	5	10,9
Le fait d'être allé chez le tradi-praticien ou personnel médical	4	8,7
Mauvais état de route	4	8,7

Il est a noté que le délai entre l'arrivée et le premier élément de prise en charge des patients était moins d'une heure chez 45 patients (97,8%). La durée entre l'arrivée et la prise des paramètres était inférieur à 5 minutes chez 30 patients (65,2%); la consultation médicale était entre 5 et 15 minutes chez 28 patients (60.9%); la prise de la première voie veineuse supérieure à 1 heure chez 24 patients (52,2%); et la paye des examens était généralement supérieure à 1 heure (78,3%) ; et la réalisation des examens était supérieure à 1 heure chez tous les patients. Certains éléments ont constitué de barrière à la prise en charge des patients et les plus observés étaient l'indisponibilité des examens prescrits et les problèmes financiers (Tableau 4). Le scanner fait au cours du suivi des patients a confirmé 11 cas d'AVC hémorragique soit 23,9% et 35 cas d'AVC ischémique soit 76,1%. Quel que soit le type d'AVC, les femmes étaient les plus atteinte avec 58,7% des cas diagnostiqués. La prise en charge hospitalière était principalement médicamenteuse (injections et perfusions) accompagnée de la kinésithérapie chez tous les patients (Tableau 5). Les plaintes des patients les plus fréquents au cours de leur suivi étaient l'asthénie chez 30 patients (65,2%) ; l'arythmie cardiaque chez 28 patients (60,9%); l'hyperthermie chez 27 patients (58,7%); l'hypotonie gauche chez 24 patients (52,2%). (Tableau 6). L'on a noté différents syndromes que présentaient les patients au cours de leur suivi hospitalier, le plus fréquent était le syndrome pyramidal observé chez 43 patients (93,5%) (Tableau 6).

**Tableau 4:** Gestion hospitalière du patient

Variable	Effectif	Fréquence (%)
**Délai entre l’arrivée et le premier élément de prise en charge**		
< 1h	45	97,8
>1h	1	2,2
**Nature du premier contact médical**		
Médecin	18	39,1
Infirmier	16	34,8
On ne sait pas	12	26,1
**Durée entre l’arrivée et la prise des paramètres**		
< 5 min	30	65,2
5 - 15 min	14	30,4
16 - 30 min	1	2,2
31 - 60 min	1	2,2
**Durée entre l’arrivée et la consultation médicale**		
< 5 min	16	34,8
5 - 15 min	28	60,9
16 - 30 min	1	2,2
> 60 min	1	2,2
**Durée entre l’arrivée et l’administration des produits**		
16 -30 min	1	2,2
31 - 60 min	34	73,3
> 60 min	11	23,9
> 1 h	1	2,2
**Durée entre l’arrivée et la prise de la première voie veineuse**		
16 - 30 min	1	2,2
31 min - 60 min	21	45,7
> 60 min	24	52,2
**Durée entre l’arrivée et la Paye des premiers examens prescrits**		
31 - 60 min	10	21,7
> 60 min	36	78,3
Durée entre l’arrivée et la réalisation des premiers examens prescrits	46	100
Barrière à la prise en charge du patient	33	71,7
Indisponibilité des médicaments	6	13
Indisponibilité des Ressources Financières	15	32,6
Préoccupations culturelles	3	6,5
Indisponibilité des examens prescrits	20	43,5
Indisponibilité des lits d’hôpitaux	2	4,3

**Tableau 5:** Soins administrés aux patients

Variable/Statistique au cours des 72 premières heures	Effectif	Fréquence (%)
**Kinésithérapie** (Oui)	46	100
**Nombre séances prescrites**		
0	6	13
1	13	28,3
2	22	47,8
3	5	10,9
**Nombre Séances Fait**		
0	13	28,3
1	14	30,4
2	19	41,3
**Injection** (Oui)	46	100
**Fréquence prescrite**		
2 fois/jr	5	10,9
3 fois/jr	40	87
> 3 fois/jr	1	2,2
**Nombre Prise prescrite**		
3	1	2,2
6	1	2,2
9	42	91,3
12	2	4,3
**Nombre soins Fait**		
3	1	2,2
6	1	2,2
9	42	91,3
12	2	4,3
**Perfusion** (oui)	46	100
**Fréquence prescrite**		
1 fois/jr	3	6,5
2 fois/jr	43	93,5
**Nombre Prise prescrite**		
2	2	4,3
4	13	28,3
6	31	67,4
**Nombre soins Fait**		
2	2	4,3
4	13	28,3
6	31	67,4

**Tableau 6:** Principales plaintes et caractéristiques cliniques des patients

Plaintes des patients	Effectif	Fréquence (%)
Asthénie	30	65,2
Arythmie cardiaque	28	60,9
Hyperthermie	27	58,7
Hypotonie gauche	24	52,2
Hémiparésie gauche	23	50
Céphalées	23	50
Hypoesthésie gauche	23	50
Hémiparésie droite	21	45,6
Hypotonie droite	21	45,6
Hypoesthésie droite	21	45,6
Dysarthrie	17	40
Syndrome pyramidal	43	93,5
Syndrome Métabolique	26	56,5
Syndrome de paralysie Faciale	20	43,5
Syndrome Confusionnel	17	37
Syndrome coronarien	13	28,3
Syndrome de condensation pulmonaire	6	13
Syndrome méningé	3	6,5

L'étude a montré qu'au cours des premières 24 heures, l'état de 28 patients (60,9%) s'était aggravé et aucun des patients n'avait repris son autonomie. Au cours des 72 premières heures, 24 des patients (52,2%) qui avaient perdus connaissance avaient une amélioration de leur état de conscience; et dans l'ensemble, 25 patients (54,3%) avaient un état stable. A la sortie après plus de 10 jours d'hospitalisation pour tous les patients, 39 patients (84,8%) avaient une amélioration nette de leur état de conscience; 32 (69,6%) avaient repris leur autonomie et on avait 5 patients décédés (10,9%) et 14 (30,43%) avaient des complications à type d'hémiplégie (Tableau 7).

**Tableau 7:** Description de l’évolution clinique des patients

Evolution clinique	Après 24h		Après 72h		Après 10 jours		A la sortie après 10 jours d’hospitalisation	
	Effectif	Fréquence (%)	Effectif	Fréquence (%)	Effectif	Fréquence (%)	Effectif	Fréquence (%)
Amélioration avec reprise de conscience	8	17,4	24	52,2	40	87	39	84,8
Amélioration avec reprise de l’autonomie	0	0	2	4,3	22	47,8	32	69,6
Amélioration globale	8	17,4	36	78,3	41	89,1	40	87
Stable	17	37	25	54,3	13	28,3	2	4,3
Aggravé	28	60,9	7	15,2	3	6,5	1	2,2
Décédé	0	0	0	0	3	6,3	5	10,9
Va mieux sortie	0	0	0	0	0	0	40	87

## Discussion

La principale limite de notre étude était celle du biais d'information qui relève du fait que certains patients ne pouvaient pas se souvenir de certaines informations notamment sur la gestion pré hospitalière à cause des pertes de connaissances. Les accidents vasculaires cérébraux sont devenus un problème de santé publique dans les pays en développement car ils sont non seulement fréquents et causent d'une mortalité importante, mais ils induisent également un coût et une charge pour les familles et la société du fait des séquelles et du handicap chez les survivants. L'objectif général de cette étude était de décrire la distribution de l'évolution et de l'itinéraire thérapeutique des patients reçus pour accident vasculaire cérébral à l'hôpital régional de Bafoussam. Les patients inclus dans l'étude étaient âgés de 42 à 88 ans avec une moyenne d'âge à 62 ans. L'âge est un facteur déterminant dans la survenue d'AVC, car la majorité de nos patients était âgée de plus de 50 ans, soit 84.9% des patients; ces chiffres concordent avec ceux déjà trouvé à Bamako en 2010 par Nana Camara [8]. Les femmes étaient les plus atteint avec 27 cas soit 58,7% des AVC diagnostiqués. Ces chiffres sont semblables à ceux retrouvés par Kouakou *et al.* en 2015 à Abidjan [9]. Les ménagères étaient les plus représentées avec 23 patients (50%). Ce chiffre étant différent de celui trouvé par Nana Camara à Bamako en 2010 [8] où les fonctionnaires étaient les plus représentés avec 37,1% des cas. Cette différence peut venir du fait que la majorité des patients de son étude étaient essentiellement des résidents d'un milieu purement urbain. L'hypertension artérielle étant le principal facteur de risque des AVC, il a été retrouvé chez 22 (47,8%) patients. L'étude a également montré que 13 des patients (37,1%) étaient diabétique. Ces chiffres élevés concordent avec ceux retrouvé par Sagui en 2007 [10] après une revue documentaire des études menées en Afrique subsaharienne sur les AVC qui étaient respectivement de 68,5% et 37,3%.

Au total 46 patients victimes d'AVC ont été inclus dans l'étude parmi lesquels 9 patients (19,6%) sont venus à l'HRB pour leur première consultation médicale et 80,4% de cas référé d'une autre FOSA. Cela s'expliquerait par le fait que l'HRB dispose d'un plateau technique suffisant pour une prise en charge efficace et complète des cas d'AVC. Le mode d'apparition des symptômes était progressif chez la majorité des patients soit 45,7% et les symptômes les plus observé ayant incités à la consultation sont les céphalées (67,4%), l'asthénie (91,3%), la perte de connaissance (87%) et la faiblesse de l'hémicorps gauche (58,7%). Ces symptômes ont également été les plus fréquents à Abidjan en 2015 par Kouakou *et al.* [9] même si elles étaient à des proportions nettement inférieures. Ces fortes proportions s'expliqueraient par le fait que ce sont les principaux symptômes qui militent très souvent en faveur d'un diagnostic d'AVC. Les personnes présentant ces symptômes doivent consulter immédiatement. Le délai entre la survenue et la première consultation médicale était compris entre 1-5h avec une proportion de 63% des patients reçus. Le temps est un facteur crucial et le délai long de prise en charge est un facteur compromettant le pronostic. Ce délai ne doit pas dépasser 3 heures. Les cas d'AVC enregistré étaient au nombre de 46 dont 11 cas d'AVC hémorragique soit 23,9% et 35 cas d'AVC ischémique soit 76,1%. Quel que soit le type d'AVC, les femmes étaient les plus atteint avec 58,7% des cas diagnostiqués. Ces résultats sont proches de ceux trouvés dans de ceux publiés en 2015 par l'OMS et de l'étude de Kouakou *et al.* en 2015 à Abidjan [9, 11].

La kinésithérapie était prescrite chez tous les patients, beaucoup plus à une fréquence de 1 fois/jr. Le nombre de séances prescrites et faite au cours des 72 premières heures était beaucoup plus de 2 fois/jr. La rééducation précoce à la phase aiguë des AVC améliore le pronostic des patients. Le taux de récupération post AVC est fonction de nombreux facteurs et il est impossible de présager du recouvrement des différentes fonctions au stade aigu. La caractéristique clinique la plus fréquente était le syndrome pyramidal observé chez 93,5% des patients. La majorité des patients avaient une amélioration de leur état pendant les 10 premiers jours d'hospitalisation. Néanmoins, Le suivi hospitalier a donné une incidence de décès de 5 sur 46 (10,9%) patients reçus pour AVC; ce chiffre est inférieur aux résultats trouvé à Douala en 2016 par Kuaté-Tegueu *et al.* [12]. Cette différence s'expliquerait par le temps de suivi des patients de notre étude qui était relativement court.

## Conclusion

Au sorti de cette étude qui avait pour objectif de décrire la distribution de l'évolution et de l'itinéraire thérapeutique des patients reçus pour AVC à l'hôpital régional de Bafoussam. Il en ressort que 46 patients étaient inclus dans cette étude avec un âge moyen à 62 ans, avec un sexe ratio homme/femme de 0,7 (IC à 95%). Peu de patients recourent aux soins non médicalisés avant l'arrivée à l'hôpital. La plupart bénéficient des soins dans les délais requis mais les taux de complications et de décès hospitaliers restent élevés. Au regard de ces résultats, Il y'a une nécessité d'améliorer la communication sur l'importance non seulement des signes annonciateurs des AVC, mais aussi sur l'importance d'une consultation précoce dès l'apparition de ces signes et symptômes révélateurs, qui sans doute influencerait grandement sur le pronostic du patient. Une étude devrait être faite pour déterminer les facteurs contribuant à un taux élevé de complications et de décès chez les patients hospitalisés pour AVC à l'hôpital régional de Bafoussam.

### Etat des connaissances actuelles sur le sujet

- Fréquence élevée des cas d'AVC au sein de la population Camerounaise due à une prise en charge inadéquate des cas;
- La disponibilité des personnes qualifiées pour la prise en charge des facteurs de risque est limitée et le système de détection des cas n'est pas suffisamment sensible.

### Contribution de notre étude à la connaissance

- Documenter les faiblesses sur les conditions de transport, les délais de prise de décision pour la recherche des soins, les sources de soins et les réponses offertes par ces sources;
- Les attitudes du personnel de santé et des proches des personnes malades quand surviennent les cas d'AVC.

## Conflits d’intérêts

Les auteurs ne déclarent aucun conflit d'intérêts.

## References

[1] OMS (2019). Themes de santé: accident vasculaire cérébral (AVC).

[2] Lindsey MP, Gubitz G, Bayley M, Hill MD, Davies-Schinkel C, Singh S (2010). Canadian best practice recommandations for stroke care: on behalf of the canadian stroke strategy best practices and standards writing group.

[3] Tandi TE, Cho Y, Akam AJ, Afoh CO, Ryu SH, Choi MS (2015). Cameroun public health sector: shortage and inequalities in geographic distribution of health personnel. Int J Equity Health.

[4] Nghemkap A (2016). Cameroun: comment comprendre et prévenir les AVC dans notre contexte actuel.

[5] Rusinaru M (2010). Identification et prévalence des facteurs de risque de l'accident vasculaire cérébral en médecine générale: enquête rétrospective dans une unité de soins, d'enseignement et de recherche de médecine ambulatoire en lorraine, de 2000 à 2010, et comparaison à l'étude interstroke.

[6] Jingi AM, Noubiap JJ (2015). Cardiovascular risk factors awareness and prevalence among primary care physician: an insight from the West region Awareness Initiative Surgery to fight cardiovascular disease (WAIT-CVD) in Cameroon. BMC Res Notes.

[7] Lkoubou A, Awah P, Fezeu L, Sobngwi E, Kengne AP (2010). Hypertension, diabetes mellitus and task shilling in their management in sub-saharan Africa. Int J Environ Res Public Health.

[8] Nana C (2010). Prise en charge des accidents vasculaires cérébraux en réanimation polyvalente au CHU du point G.

[9] Kouakou Y, Traore F, Tano M, Kakou AJB, Konin C, Kakou GM (2015). Aspects épidémiologiques des accidents vasculaires cérébraux (AVC) aux urgences de l'institut de cardiologie d'Abidjan. The pan African medical journal.

[10] Sagui E (2007). Les accidents vasculaires cérébraux en Afrique subsaharienne. Med trop.

[11] OMS (2015). Que faire pour éviter une crise cardiaque ou un accident vasculaire cérébral?.

[12] Kuate-Tegueu C, Mapoure NY, Gopdjim ML, Doumbé J, Noubissi-Dada G, Dissongo J (2016). Mortalité par accident vasculaire Cérébral et ses Déterminants dans un Hôpital de Référence de Douala (Cameroun). Health Science and Diseases.

